# Atrial fibrillation caused by *Daboia palestinae* snakebite: a case report

**DOI:** 10.1093/omcr/omad136

**Published:** 2023-12-19

**Authors:** Marah Khaldy, Hasan Arafat, Yasmina Khaldi

**Affiliations:** Department of Internal Medicine, Augusta Victoria Hospital, Jerusalem, State of Palestine; Department of Internal Medicine, Augusta Victoria Hospital, Jerusalem, State of Palestine; Faculty of Medicine, American Arab University, Jenin, State of Palestine

**Keywords:** snake envenomation, arrhythmia, cardiotoxicity, Palestine viper

## Abstract

**Background:**

snake envenomation is a serious healthcare issue. *Daboia palaestinae* is an endemic species to the Middle East that is responsible for the majority of envenomation cases with serious health issue consequences.

**Case Presentation:**

we report a case of a 20-year-old Palestinian man who presented to emergency room following a snake bite. He developed atrial fibrillation which is a rare but serious complication of *D. palaestinae* snakebite. We reviewed the proper approach and management to such cases.

**Conclusion:**

cardiac arrhythmias are a rare but serious, often fatal, complication of snake envenomation. Early detection and proper management is key to avoid morbidity and mortality.

## INTRODUCTION

*Daboia palaestinae (Dp)* is an endemic, venomous species of snakes endemic to Palestine and the surrounding region [1]. Without immediate care, its bite can be life-threatening and result in substantial mortality and morbidity [2]. The severity and the venomous activity is the result of various proteins and enzymes, usually leading to increased capillary permeability, endothelial damage, and disruption of the coagulation system leading to hemorrhagic activity and edema. Myotoxicity, myonecrosis, and cardiotoxicity were also reported as less likely effect of envenomation [3].

Here we present a case of a 20-year-old Palestinian male presenting with atrial fibrillation following a snake bite by *Dp.*

## CASE PRESENTATION

**Figure 1:**
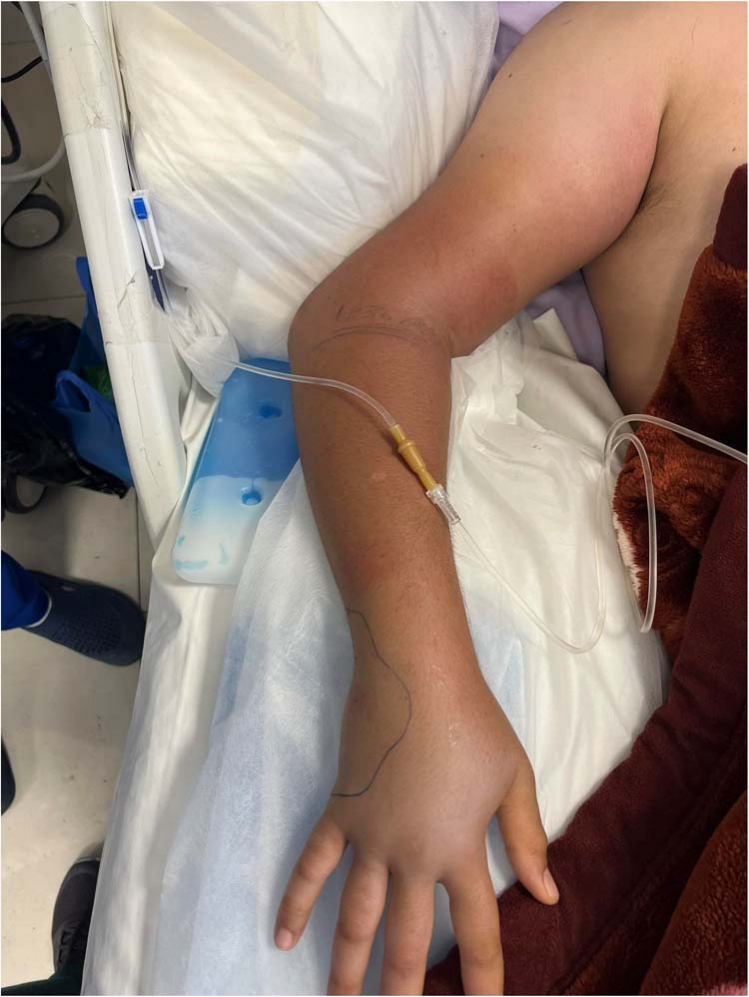
Right upper limb showing the bite site, marked. Swelling and tenderness extending from the site of the bite at the ulnar aspect of the dorsum of the hand to the elbow joint.

**Figure 2:**
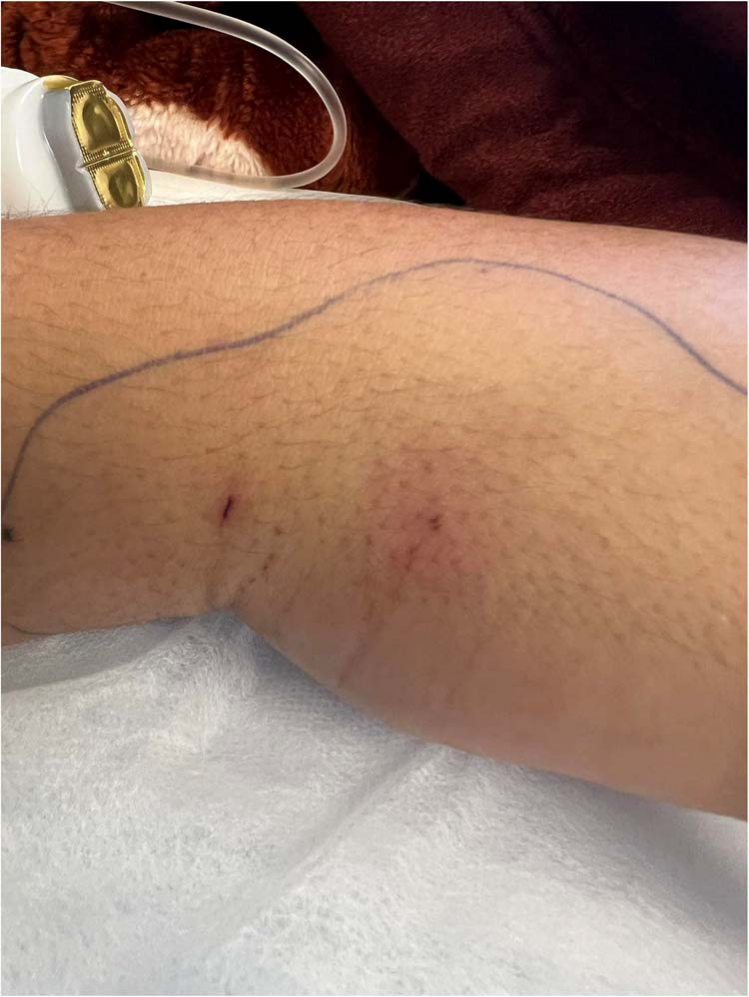
Puncture wounds resulting from snake bite.

A 20-year-old male patient with a free past medical and surgical history, presented to emergency room (ER) complaining of right sided hand swelling after sustaining a snake bite. The bite was associated with severe pain and numbness at the back of his hand, in addition to nausea, vomiting and diffuse sweating. He had a blood pressure of 100/60 mmHg, a heart rate of 120 beats per minute (bpm), a temperature of 36.6 degrees, and an O_2_ saturation of 90% on room air. He was sweaty and anxious, his right hand, shown in fig. 1, was edematous, nearly doubling in size compared to the left side with two obvious puncture wounds as seen in fig. 2. These findings were associated with ecchymosis and tenderness to palpation extending from the dorsum of the hand to above the elbow joint. Range of motion was limited at the wrist joint, while the elbow joint had a full range of motion. Sensation was intact and both the radial and brachial pulses were full and palpable. Otherwise, his examination was unremarkable. Electrocardiogram (ECG) showed sinus tachycardia, mostly related to stress (shown in fig. 4). Complete blood count (CBC) showed a hemoglobin level of 15.8 mg/dl, white blood cell count was 13 300, platelet count was 430 000. Prothrombin time (PT) was 14.3, activated partial thromboplastin time (aPTT) was 25, international normalized ratio (INR) was 1.1.

**Figure 3:**
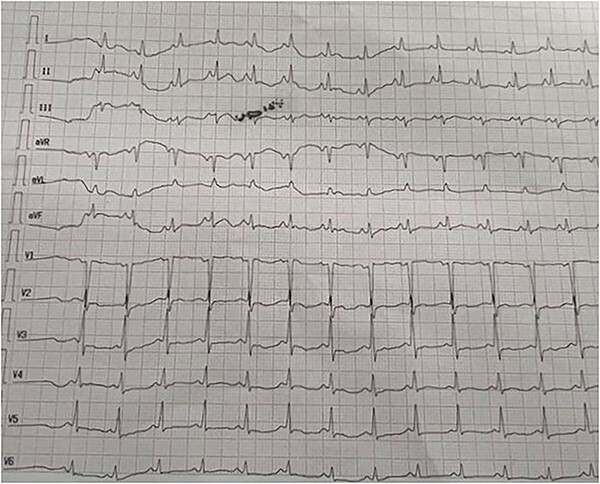
The snake responsible for the bite, identified as *Daboia palaestinae*.

The snake was killed and brought to the ER, it was identified as the Palestine viper, known by its scientific name *D. palaestinae*. The snake is shown in fig. 3.

**Figure 4:**
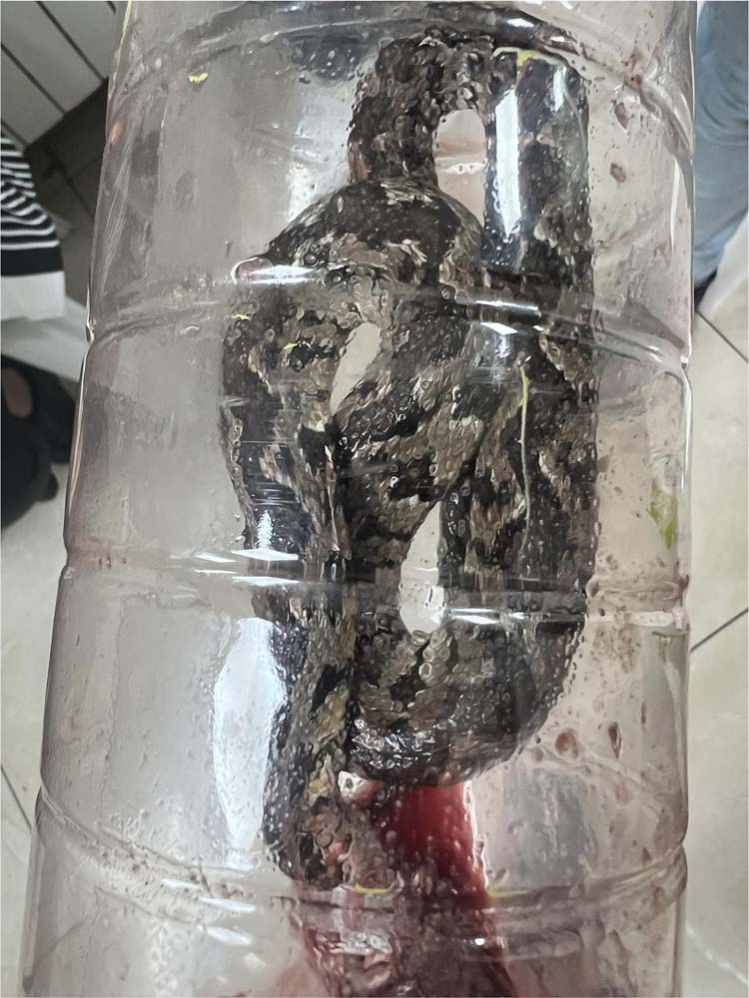
Electrocardiogram at presentation. The rhythm here shows sinus tachycardia, mostly related to stress.

**Figure 5:**
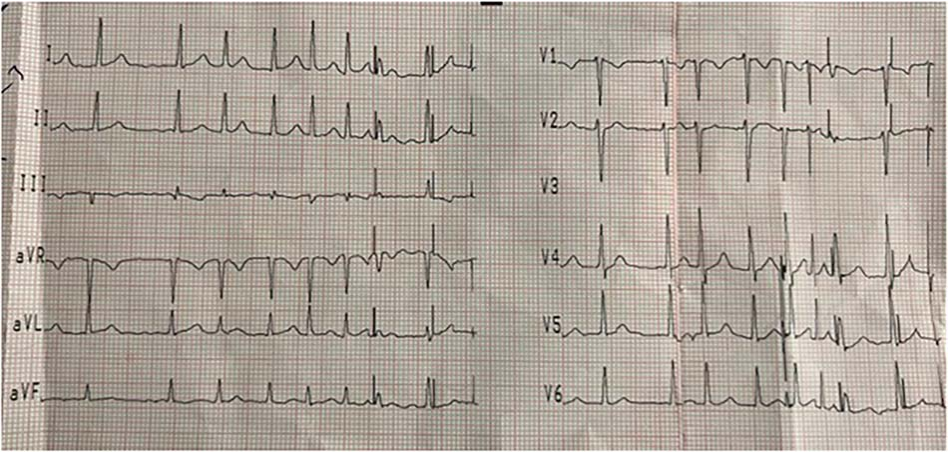
Electrocardiogram showed an irregularly irregular rhythm with absent P-waves, suggestive of atrial fibrillation.

The patient received eight vials of polyvalent anti-venom while in the emergency room, after which he reported significant improvement in terms of pain. He was admitted to intensive care unit (ICU) for close monitoring. On the second day of admission, the patient developed sudden onset palpitation associated with shortness of breath without chest pain. His heart rate was 155 bpm, blood pressure 130/65 mmHg, O2 saturation 95%, temperature 36.6. CBC, obtained during morning routine labs prior to the episode, showed the following findings: hemoglobin: 14.4 mg/dl, white blood cell count: 17 500, platelet count 355 000. Other labs, including a comprehensive metabolic panel, lactate dehydrogenase (LDH): 243. PT: 14.7, INR: 1.3, aPTT: 28, fibrinogen 271 mg/dl. His troponin I peaked at 0.04 ng/ml (Reference range < 0.029), creatine kinase-muscle/brain was 0.3% (reference range 0–25). Electrocardiogram (ECG), shown in fig. 5, showed irregularly irregular pulse with absent P-wave, these changes were absent on the ECG done on admission, as it showed a sinus, regular heart rate (shown in fig. 4). The picture was suggestive of new-onset atrial fibrillation. Transthoracic echocardiography noted normal biventricular function with no focal wall motion abnormality, and no valvular heart diseases with normal ejection fraction.

As the patient had a free past medical history, review of medications did not disclose any medication that could incite such an event, no previous episodes within the patient known relatives was reported by the patient. The new onset cardiovascular findings were considered part of the disease process, and a manifestation of ongoing venom toxicity. Based on that interpretation, six more vials of polyvalent anti-venom were administered.

Cardiology team was consulted and the patient was started on bisoprolol 5 mg per os (PO) once daily (OD), which failed to control his rate. He was given amiodarone 300 mg STAT then kept on continuous infusion of 900 mg over 24 h. The patient was monitored for any new onset hematologic or cardiac events via serial CBC, INR, fibrinogen and troponin I every 6 h.

**Figure 6:**
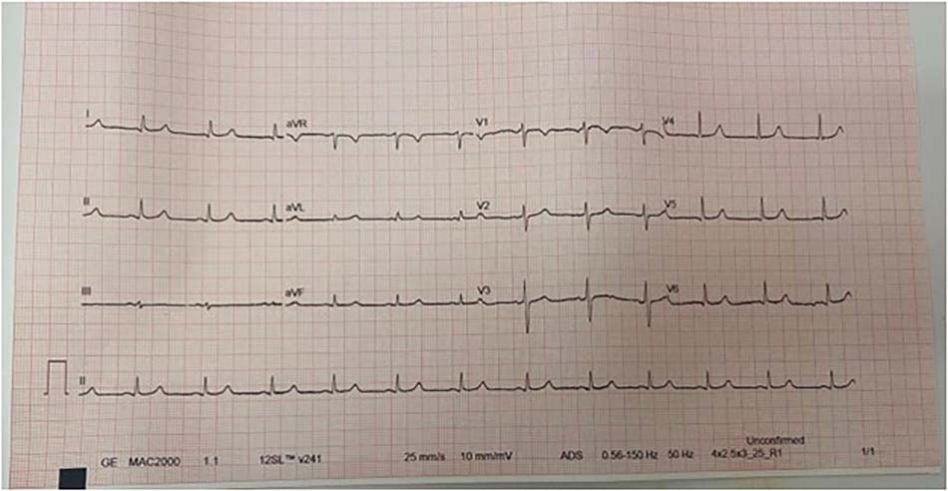
Electrocardiogram following resolution of the event, it shows a sinus rhythm with a heart rate of 76 bpm.

The patient’s cardiac enzymes improved the next day, CBC parameters and coagulation profile remained within normal. After 24 h of monitoring, in which the patient had no new concerning events, the patient was transferred to the medical ward. ECG next day showed normal sinus rhythm with ventricular rate of 76 bpm (shown in fig. 6). Left upper limb swelling improved over the following three days, and the patient was discharged home on oral amoxicillin-clavulanate for 7 days.

He was seen in the outpatient clinic 5 weeks later, the swelling of his upper limb had completely resolved, he reported no new episodes of palpitation of chest discomfort. CBC, basic metabolic panel, and coagulation profile were within normal limits. New echocardiography showed no abnormalities. The patient was maintained on bisoprolol, 5 mg PO OD. As no new episodes were reported, no further investigations were requested as the entire episode was deemed secondary to snake envenomation, which had completely resolved by the time of follow up.

## DISCUSSION

Snake envenomation is considered a major public health issue worldwide. 4.5–5.4 million people fall victims to snakebites every year [4]. *Dp,* a ubiquitous, venomous viper species endemic to the Eastern Mediterranean region, accounts for the majority of snakebites in the region. Due to its arsenal of enzymatic polypeptides, which exert pernicious vascular, neurotoxic and hemostatic effects, the clinical outcome following envenomation can be quite variable, depending on the anatomic location and amount of venom delivered. Localized signs are often limited to pain, hemorrhage, edema and sometimes regional lymphadenopathy. In more severe cases, bleeding, cardiac arrhythmias, and even shock can ensue [5]. Hemorrhage is the main cause of death following *Dp* envenomation [6], however, cardiac toxicity has been also documented in *Dp* victims, this was more observed in individuals with underlying co-morbidities (diabetes, ischemic heart disease, hypertension, dyslipidemia and advanced age). Patients might be asymptomatic, or they may develop chest pain, palpitation, and hypotension. Myocardial infarction was reported in adult males from tropical regions with free past medical history. ECG dysfunction due to *Dp* can be due to direct or indirect effects of the venom. While direct effects result from alteration of the action potential of cardiac cell membranes, or changes in intracellular ion activity, indirect effects develop in response to hypotension [4, 7]. Sinus tachycardia is the most commonly seen change, it is observed in the setting of hypovolemia due to extravasation of fluid in the affected limb, or due to hypotension predisposed by anaphylaxis [8]. Atrial fibrillation, on the other hand, has been rarely reported in the setting of *Dp* envenomation.

Wang A et al reported a case of myocarditis following *Dp* snake bite without specifying if any ECG changes were observed [7]. Sogut O et al reported a case of atrial fibrillation following a snake bite of unknown species, however, the effect was attributed to epinephrine administered subcutaneously due to anaphylaxis developing secondary to antivenom administration rather than the venom itself [9]. Thillainathan S et al reported a case of *Hypnale hypnale* bite resulting in ECG changes suggestive of atrial fibrillation and inferior wall myocardial infarction. The episode was attributed to coronary artery spasm at it resolved spontaneously [10].

Although not well understood, myotoxicity is attributed to two major classes of snake venom toxins. The first class is phospholipases, which exhibit numerous pharmacological effects, including myotoxicity, neurotoxicity and cytotoxicity, with the other class being L-amino acid oxidases (LAAO), which are flavoproteins that function by catalyzing the stereospecific oxidative deamination of L-amino acid to α-keto acids. This class exerts direct cytotoxic activity, leading to edema, hemorrhage, myotoxicity and apoptosis [1]. The cause of specific arrhythmias and ECG changes is poorly understood. Several mechanisms have been hypothesized to address the issue: (1) direct cytotoxic effect (2) toxin-induced arrhythmia (3) acute coronary syndrome secondary to coagulopathy (4) toxin-mediated coronary spasm (5) hyperkalemia secondary to acute renal failure and (6) inflammatory processes secondary to venom-induced hypersensitivity [11].

**Figure 7:**
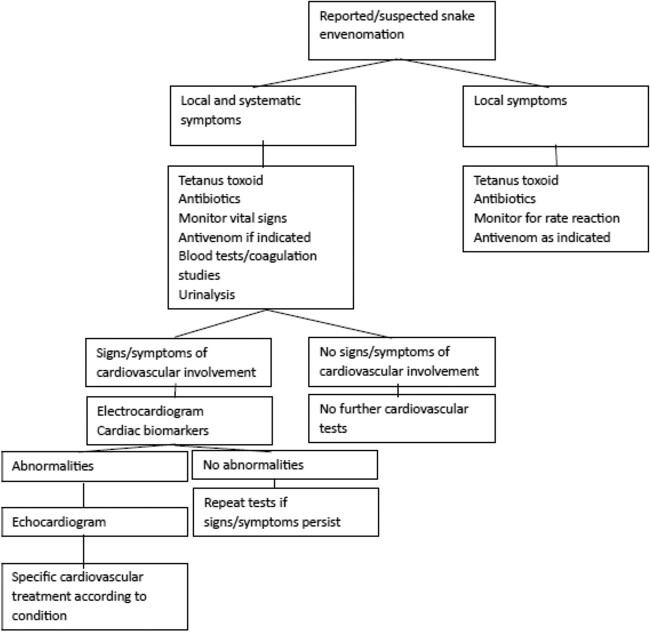
Proposed approach for snake envenomation associated with cardiovascular abnormalities [11].

Treatment of viper envenomation consists of supportive care and immobilization of the affected extremity. Tourniquets, compression dressing, and attempts to remove the venom by cutting or suction should be discouraged. Coagulopathy and hemorrhage are common complications of *Dp* envenomation. All patients should be evaluated with hemoglobin, platelet count and fibrinogen. Polyvalent antivenom remains the treatment of choice for viper envenomation [8]. Patients should be monitored due to significant risk of allergic reaction (20% of cases). Tetanus toxoid and antibiotics can be administered if there is high-risk of infection [11]. Patients who develop ECG dysfunction often require more aggressive measures, including respiratory support via mechanical ventilation for those who develop respiratory failure. More specific measures include administering additional doses of antivenom to neutralize snake venom and reduce cardiac dysfunction. Lidocaine, amiodarone, and beta blockers can be used against cardiac arrhythmias [4]. A proposed algorithm is shown in fig. 7.

## CONCLUSION

Cardiac arrhythmia is a serious, often fatal, complication of snake envenomation. Screening for arrhythmia, conduction disorders and other cardiac-related issues, particularly in the setting of severe envenomation, can prevent further complication and death.

## Data Availability

The data used in this case report is available upon reques from the corresponding author.
